# Imetelstat (a telomerase antagonist) exerts off-target effects on the cytoskeleton

**DOI:** 10.3892/ijo.2013.1865

**Published:** 2013-03-27

**Authors:** ILGEN MENDER, SERIF SENTURK, NURIMAN OZGUNES, K. CAN AKCALI, DIMITRIS KLETSAS, SERGEI GRYAZNOV, ALP CAN, JERRY W. SHAY, Z. GUNNUR DIKMEN

**Affiliations:** 1 Department of Biochemistry, Faculty of Medicine, Hacettepe University, Ankara, Turkey;; 2 Department of Molecular Biology and Genetics, Bilkent University, Ankara, Turkey;; 3 Department of Histology and Embryology, Faculty of Medicine, Ankara University, Ankara, Turkey;; 4 Laboratory of Cell Proliferation and Ageing, Institute of Biology, National Centre for Scientific Research ‘Demokritos’, Athens, Greece;; 5 Geron Corporation, Menlo Park, CA;; 6 University of Texas Southwestern Medical Center, Dallas, TX, USA;; 7 Center of Excellence in Genomic Medicine Research, King Abdulaziz University, Jeddah, Saudi Arabia

**Keywords:** GRN163L, cell adhesion, E-cadherin, matrix metalloproteinase-2, non-small cell lung cancer

## Abstract

Telomerase is a cellular ribonucleoprotein reverse transcriptase that plays a crucial role in telomere maintenance. This enzyme is expressed in approximately 90% of human tumors, but not in the majority of normal somatic cells. Imetelstat sodium (GRN163L), is a 13-mer oligonucleotide N3′→P5′ *thio*-phosphoramidate lipid conjugate, which represents the latest generation of telomerase inhibitors targeting the template region of the human functional telomerase RNA (hTR) subunit. In preclinical trials, this compound has been found to inhibit telomerase activity in multiple cancer cell lines, as well as *in vivo* xenograft mouse models. Currently, GRN163L is being investigated in several clinical trials, including a phase II human non-small cell lung cancer clinical trial, in a maintenance setting following standard doublet chemotherapy. In addition to the inhibition of telomerase activity in cancer cell lines, GRN163L causes morphological cell rounding changes, independent of hTR expression or telomere length. This leads to the loss of cell adhesion properties; however, the mechanism underlying this effect is not yet fully understood. In the present study, we observed that GRN163L treatment leads to the loss of adhesion in A549 lung cancer cells, due to decreased E-cadherin expression, leading to the disruption of the cytoskeleton through the alteration of actin, tubulin and intermediate filament organization. Consequently, the less adherent cancer cells initially cease to proliferate and are arrested in the G1 phase of the cell cycle, accompanied by decreased matrix metalloproteinase-2 (MMP-2) expression. These effects of GRN163L are independent of its telomerase catalytic activity and may increase the therapeutic efficacy of GRN163L by decreasing the adhesion, proliferation and metastatic potential of cancer cells *in vivo*.

## Introduction

Non-small cell lung cancer (NSCLC) is the most common cause of cancer-related mortality. Doublet combination chemotherapy is currently the first-line therapy for advanced lung cancer that is not surgically resectable. However, even with chemotherapy, the prognosis of patients with advanced NSCLC remains poor, with a 1-year survival rate of 30% (1,2). Therefore, it is important to develop new treatment regimens in order to reduce the morbidity and mortality of this fatal disease.

Telomeres are DNA repeats (TTAGGG)_n_ found at the end of chromosomes, and play an important role in maintaining genomic stability (3,4). Due to the end replication problem, telomeres are progressively lost with each cell division, eventually leading to cell growth arrest (replicative senescence) in normal cells (5,6). Critical telomere shortening may be considered as an initial block to indefinite cellular proliferation (a hallmark of cancer). However, telomere shortening may be counteracted by the cellular ribonucleoprotein reverse-transcriptase telomerase (hTERT), which uses a part of an internal RNA moiety as a template for the synthesis of telomeric repeats (7,8). Telomerase activity is not readily detectable in most quiescent normal somatic tissues; it is, however, highly expressed in ∼90% of human tumors. This feature renders this enzyme an attractive, almost universal, target for cancer therapy. Therefore, various telomerase inhibitors have been developed over the past few years (9,10). Among these compounds, a *thio*-phosphoramidate oligonucleotide, imetelstat sodium (GRN163L), is being assessed in clinical trials as a potent human telomerase inhibitor. This molecule was designed as a competitive telomerase inhibitor, which binds directly to the active site of the enzyme, thus inhibiting its activity. The presence of the covalently conjugated 5′-palmitoyl (C16) lipid group provides more effective cellular uptake and increased bioavailability of GRN163L (11,12). This compound is currently in multiple phase II clinical trials as a potential broad-spectrum anticancer agent.

Our previous *in vitro* studies showed that GRN163L effectively inhibits telomerase activity in A549 lung cancer cells, reduces their proliferation rate within 3–4 weeks and progressively shortens telomere length in 5–6 weeks, leading to apoptotic cell death. Moreover, GRN163L effectively inhibits the formation and growth of lung metastases in xenograft animal models *in vivo*(13). We have also reported that A549 cells treated with a single dose of GRN163L (1 *μ*M) prior to cell attachment, were relatively weakly attached to the plate surface substrates and were morphologically altered (i.e., became rounded), whereas mismatch (MM) control-treated cells exhibited a typical epithelioid appearance and normal adhesion properties. These morphological changes were independent of human telomerase RNA (hTR) subunit expression or telomerase inhibition and were unrelated to telomere length. We determined that these effects were due to the molecular structure of the oligo *thio*-phosphoramidate and its lipid moiety, the N3′→P5′-*thio*-phosphoramidate backbone and the presence of G-quadruplex-forming triple-G sequences within the GRN163L (14). However, the exact mechanism underlying these morphological changes remains unknown.

Microfilaments, microtubules and intermediate filaments are fundamental structures of the cytoskeleton, which play important roles in the determination of cell shape, proliferation and migration. F-actin filaments are required for cell shape determination, microtubules are responsible for the positioning of organelles playing a pivotal role in intracellular transport and intermediate filaments provide mechanical support and resistance to stress (15). Cadherins are Ca^2+^-dependent adhesion molecules. One of the most widely investigated is E-cadherin, which influences cellular shape and cell-cell interactions. The loss of E-cadherin-mediated adhesion is considered to be characteristic of the transition from benign lesions to invasive and metastatic cancer (16). In the present study, we investigated whether the cytoskeletal and cell adhesion proteins are associated with the observed rapid morphological alterations (i.e., ‘rounding effect’) and the loss of adhesion of A549 lung cancer cells treated with a single dose of GRN163L (1 *μ*M). In addition, since it has been shown that the overexpression of telomerase in cancer cells increases the level of matrix metalloproteinase-2 (MMP-2), which is directly involved in the invasion process (17), we observed that GRN163L decreased MMP-2 expression, suggesting that GRN163L exerts some of its anticancer effects in a telomere-independent manner.

## Materials and methods

### Cell culture

A549 non-small lung cancer cells were obtained from the American Type Culture Collection (ATCC; Manassas, VA, USA). A549 cells were cultured in DMEM containing 10% fetal bovine serum (FBS; Sigma, St. Louis, MO, USA) and 100 U/ml penicillin-streptomycin (Sigma). The 13-mer GRN163L (Geron Corp., Menlo Park, CA, USA), which complements the template region of telomerase hTR (also known as hTERC), and the MM control oligonucleotide, which does not complement the template region of hTR, were prepared as previously described (11).

### Western blot analysis

The A549 cells (1×10^6^) were plated in 6-well plates and immediately treated with MM (1 *μ*M) or GRN163L (1 *μ*M). The untreated control and treated cells were collected following a 24-h incubation period and lysed with NP-40 lysis buffer containing 50 mM Tris-HCl (pH 8.0), 150 mM NaCl, 1% NP-40 detergent and 1X protease inhibitor complex (Roche Applied Science, Indianapolis, IN, USA). Protein concentration was quantified using the Bradford assay (Sigma), as previously described (12). A total of 40 *μ*g of protein lysate was subjected to SDS-PAGE, followed by transfer onto polyvinylidene difluoride membranes. Blocking and antibody incubation were performed in 5% milk in PBS containing 0.2% Tween-20. Membranes were exposed to X-ray film using the ECL Plus Detection reagent (Amersham Life Science, Inc., Piscataway, NJ, USA). E-Cadherin (1:1,000), β-actin (1:400), α-actinin (1:1,000), pan-cytokeratin (1:1,000), α-tubulin (1:2,000) and calnexin antibodies (1:10,000) were obtained from Santa Cruz Biotechnology, Santa Cruz, CA, USA and used for western blot analysis. Secondary antibody (Sigma) was used in proportion 1:5000. Densitometry levels for each blot were determined using calnexin as the loading control.

### Immunohistochemistry

A549 cells (1×10^5^) were plated onto glass coverslips and immediately treated with MM (1 *μ*M) or GRN163L (1 *μ*M). The cells were fixed in 3% paraformaldehyde for α-actinin and E-cadherin, and in microtubule stabilization buffer for F-actin, αβ tubulin and cytokeratin, following a 24-h incubation period. Fluorescein phalloidin, specific to F-actin, and mouse monoclonal antibodies against αβ tubulin (Santa Cruz Biotechnology), pan-cytokeratin (isoforms 1, 4, 5, 6, 8, 10, 13, 18 and 19), α-actinin and E-cadherin (Sigma), were applied. FITC-conjugated goat anti-rabbit IgG for pan-cadherin and FITC-conjugated goat anti-mouse IgG (Jackson ImmunoResearch Laboratories, Inc., West Grove, PA, USA) for the other cytoskeletal proteins were used as secondary antibodies. All antibodies were diluted 1:100 in PBS and incubated for 90 min at 37°C in a humidified chamber. Images were examined under a Carl Zeiss LSM 510 META Confocal Laser Scanning microscope (488-nm argon ion, 543-nm green helium neon, 633-nm red helium-neon laser lines) and consecutive optical sections were recorded and used for 3D image reconstruction.

### Cell cycle analyses

Real-time PCR was used for cell cycle analyses; the A549 cells were incubated with GRN163L for 24 h or up to 1 week. Total RNA was isolated from the control cells and GRN163L-treated cells using the RNeasy mini kit (Qiagen, Inc., USA, Valencia, CA, USA), according to the manufacturer’s instructions. cDNA synthesis was performed using the DyNamo cDNA synthesis kit (Finnzymes, Espoo, Finland). Primers were designed using Primer Design sofware (version 2.0; serial number: 52017. Copyright 1990, 91; Scientific and Educational Software) (Table I). Real-time PCR was performed using SYBR-Green (Finnzymes). Samples were heated to 94°C for 5 min as the initial denaturation, followed by 40 cycles of denaturation at 95°C for 30 sec, 55°C for 30 sec, 72°C for 30 sec and annealing/extension at 75°C for 5 min. A melt curve stage was added to analyze the PCR product. Cyclophilin A was used as an internal control gene to normalize for RNA quantity. The results were analyzed using the Bio-Rad iCycler-Techgene thermal cycler.

### Determination of MMP-2 expression

The correlation between telomerase inhibition and MMP-2 expression was evaluated by real-time PCR. To determine the effect of GRN163L on MMP-2 expression, GRN163L (1 *μ*M) was added to the medium 24 h after plating. After an additional 24 h, RNA was collected for real-time PCR from the control cells and GRN163L-treated cells using TRIzol reagent (Invitrogen, Carlsbad, CA, USA). cDNA synthesis was then performed with M-MLV RT (Invitrogen). MMP-2 expression was assessed by real-time PCR which was performed using SYBR-Green (Applied Biosystems, Carlsbad, CA, USA). The primers used for real-time PCR are presented in Table II. Samples were heated to 95°C for 3 min as initial denaturation, followed by 40 cycles of denaturation at 95°C for 3 sec, 58°C for 20 sec, 72°C for 5 sec and annealing/extension at 95°C for 1 min, 55°C for 30 sec and 95°C for 30 sec. A melt curve stage was added to analyze the PCR product. GAPDH was used as the internal control gene to normalize for RNA quantity.

### Viral transduction

shRNA (0.5 *μ*g), together with 0.5 *μ*g of helper plasmids (0.2 *μ*g pMD2G and 0.3 *μ*g psPAX2) were transfected into 293FT cells with Effectene reagent (Qiagen). Viral supernatants were collected 48 h after the transfections and cleared through a 0.45-m filter. The A549 cells were transfected with viral supernatants containing 2 *μ*g/ml polybrene (Sigma) and the successfully transfected cells were selected using puromycin.

### Invasion/cell migration assay

A549 cells were treated with 1 *μ*M GRN163L for 24 h. The untreated and treated cells (1×10^5^) were then plated in Matrigel™-coated invasion chambers (BD Biosciences, San Jose, CA, USA) and processed according to the manufacturer’s instructions. shMMP-2 knockdown cells were used as a series of control cells. Chemoattractant was added to the lower chamber (below the membrane), and culture medium, containing 10% FBS, was used for the A549 cells. Cells were incubated for 22 h at 37°C, in an atmosphere of 5% CO_2_. Cells were removed from the top chamber using cotton swabs, washed, then fixed and stained with 6% glutaraldehyde and 0.5% crystal violet for 30 min. The cells that migrated through to the bottom of the membrane and stained were counted by photographing the membrane under a microscope.

## Results

### GRN163L disrupts the organization of cytoskeletal elements

In order to investigate whether the rounding effect observed within 24 h in the GRN163L-treated A549 cells is related to any changes in the cytoskeleton, the key elements of cytoskeletal proteins were investigated using western blot analysis and immunohistochemical staining techniques.

**Figure 1:**
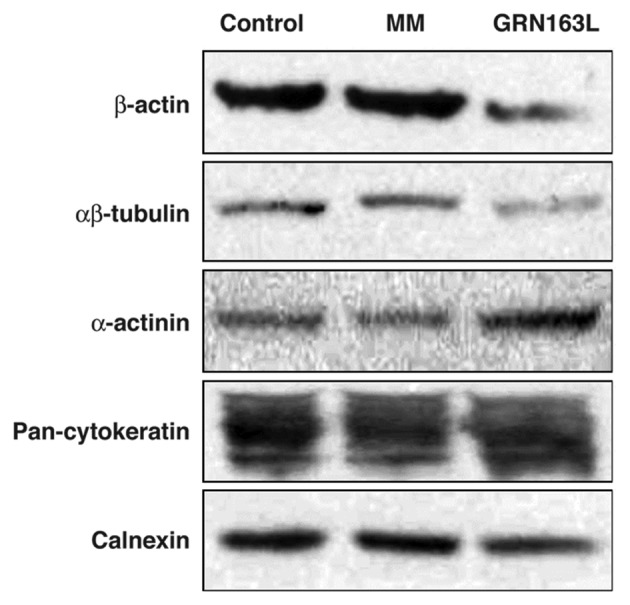
Western blot analysis of the expression of β-actin, αβ-tubulin, α-actinin and pan-cytokeratin, in A549 cells either untreated (control), or treated with GRN163L (1 *μ*M) or the mismatch (MM) (1 *μ*M) oligonucleotides. Calnexin was used as the loading control.

Actin and tubulin are the major proteins of the cytoskeleton, which determines the shape of the cell. The western blot analysis results demonstrated that actin and tubulin expression decreased following a 24-h treatment with GRN163L, when compared to the control and MM-treated cells (Fig. 1).

As an actin-binding protein, α-actinin plays multiple roles in different types of cells. In epithelial cells it is found along actin filament bundles and adherens-type junctions, where it is involved in the binding of actin to the cell membrane. We observed an approximate 2- to 3-fold increase in α-actinin expression in the cells treated with GRN163L for 24 h, compared to the control and MM-treated cells (Fig. 1).

**Figure 2:**
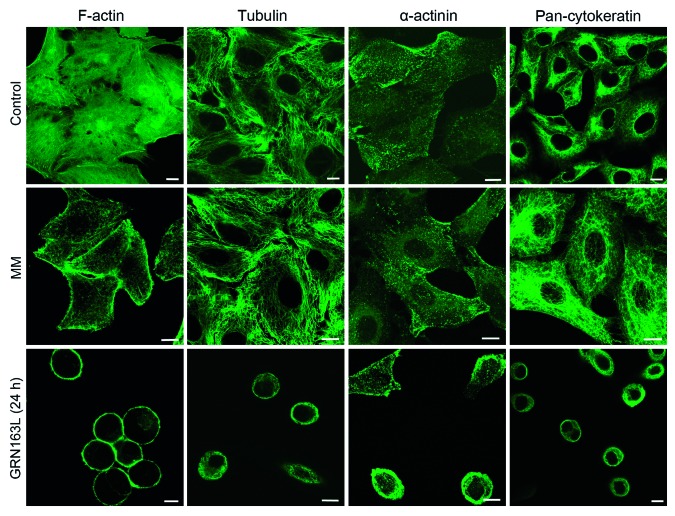
Effects of GRN163L and mismatch (MM) oligonucleotide on F-actin, tubulin, α-actinin and pan-cytokeratin filament organization in A549 cells. A549 cells were plated on chamber slides in the presence of GRN163L (1 *μ*M) and incubated for 24 h. Scale bars, 10 *μ*m.

In the untreated and MM-treated A549 cells, we observed an organization of dense actin filaments, exhibiting common cytoplasmic dispersion, as detected by immunohistochemistry analyses. GRN163L treatment prior to cell attachment disrupted the cytoplasmic distribution of actin. In the GRN163L-treated cells, essentially all the actin filaments were displaced and concentrated along the cell membrane within 24 h. These results demonstrated that the decrease in actin expression, as well as significant changes in the morphological distribution of actin in the cell, were caused by the presence of GRN163L (Fig. 2).

Similar to the intracellular distribution of actin, extensive bundles of microtubules, which ‘radiate’ throughout the cytoplasm of A549 cells, was observed in the untreated control cells. GRN163L treatment altered the perinuclear and radial organization of the tubulin cytoskeleton and microtubules were relocated toward the cell membrane, in a pattern similar to that of actin filaments (Fig. 2). The immunostaining results demonstrated that α-actinin protein was localized under the membrane, and was mostly colocalized with actin filaments (data not shown).

Intermediate filaments form homogeneous polar fibers within the cells and they are cell-type-dependent. Cytokeratins are the most common intermediate filaments found in epithelial cells. Western blot analysis of cytokeratin expression did not demonstrate any significant difference between the GRN163L-treated and control cells (Fig. 1). By contrast, immunohistochemical staining indicated that the cytokeratins were redistributed evenly throughout the cytoplasm in untreated control cells, whereas in the GRN163L-treated cells, the cytokeratins were localized to the cell periphery (Fig. 2).

**Figure 3:**
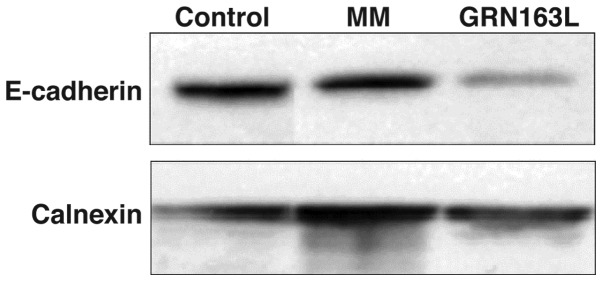
Western blot analysis of E-cadherin expression in GRN163L- (1 *μ*M) and mismatch (MM) oligonucleotide (1 *μ*M)-treated A549 cells. Calnexin was used as the loading control.

**Figure 4:**
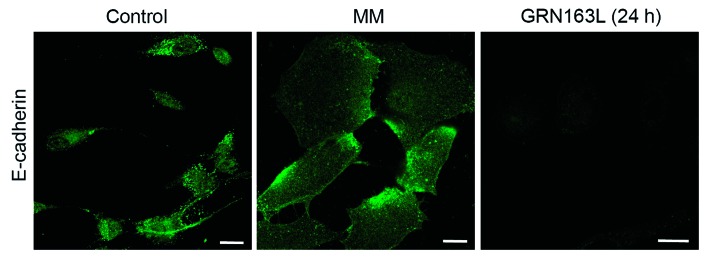
Effects of GRN163L and mismatch (MM) oligonucleotide on E-cadherin distribution. A549 cells were plated on chamber slides in the presence of GRN163L (1 *μ*M) and incubated for 24 h. Scale bars, 10 *μ*m.

Of note, GRN163L treatment resulted in significant loss of E-cadherin expression. Western blot analysis results demonstrated a significant decrease in E-cadherin expression in A549 cells within 24 h (Fig. 3). The immunohistochemical analysis results also demonstrated a decrease in E-cadherin expression at 24 h (Fig. 4).

### GRN163L does not cause morphological changes and loss of cell adhesion in the cytoskeleton after thermal denaturation (heating for 5 min at 80°C)

**Figure 5:**
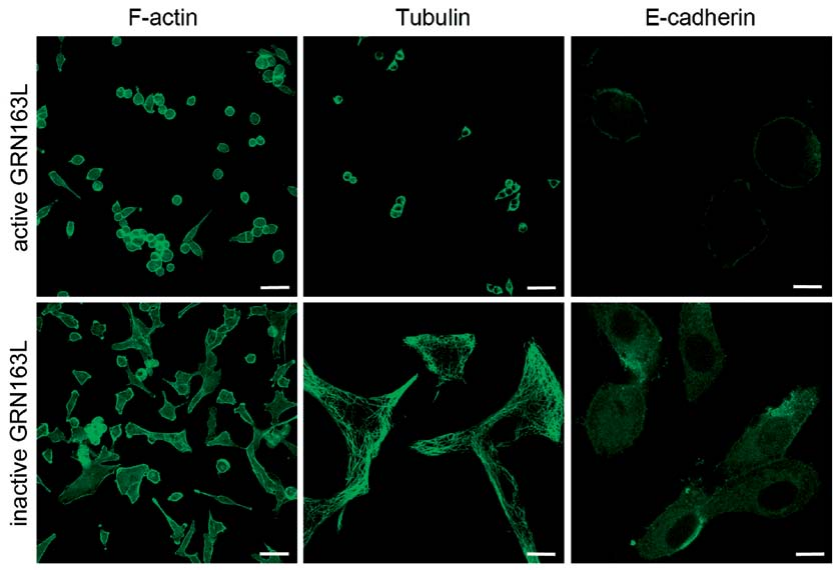
F-actin, tubulin and E-cadherin distribution in the cells treated with GRN163L and thermally-inactivated GRN163L. Scale bars, 50 *μ*m in F-actin and activated tubulin images and 10 *μ*m in E-cadherin images.

After the A549 cells were plated on coverslips, GRN163L (1 *μ*M) was heated for 5 min at 80°C, then added to the cell culture medium. Following a 24-h incubation period, the cells were fixed and stained for actin, tubulin and E-cadherin. The structures of actin, tubulin and E-cadherin were not markedly altered in the cells treated with the pre-heated GRN163L. Approximately 80% of these cells remained attached and exhibited morphological characteristics (Fig. 5) similar to the untreated control or MM-treated cells (data not shown). These results demonstrate that morphological changes and the loss of adhesion are specific to the oligonucleotide and GRN163L is ‘inactivated’ by heating for 5 min at 80°C.

### GRN163L decreases the expression of G1 phase cell cycle control genes

**Figure 6:**
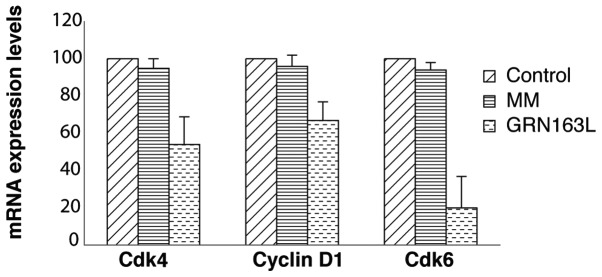
Analysis of cyclin D1, Cdk6 and Cdk4 mRNA expression in untreated, mismatch (MM) oligonucleotide- and GRN163L-treated cells by real-time RT-PCR (n=3).

The morphologically altered or ‘rounded’ cells were unable to proliferate significantly while they were exposed to GRN163L treatment during the first 72 h. To elucidate the molecular mechanism behind this initial cell cycle arrest, we evaluated the mRNA levels of cyclin D1, Cdk4 and Cdk6, which are regulators of the G1 phase of the cell cycle, by real-time PCR. After plating the cells, GRN163L was added to the medium and the cells were collected following 72 h of incubation. No significant change in Cdk4 and Cdk6 mRNA expression levels was detected following 72 h of incubation with a single dose of GRN163L (data not shown). When the second set of cells was treated twice a week and collected for cell cycle analysis, a significant reduction of cyclin D1, Cdk4 and Cdk6 mRNA expression levels was observed, compared to the untreated controls (Fig. 6).

### GRN163L treatment decreases MMP-2 expression and invasion of A549 lung cancer cells through Matrigel

**Figure 7:**
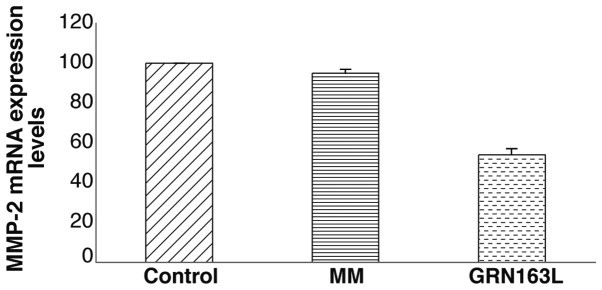
Analysis of MMP-2 mRNA expression in untreated, mismatch (MM) oligonucleotide- and GRN163L-treated cells by real-time RT-PCR (n=3).

**Figure 8:**
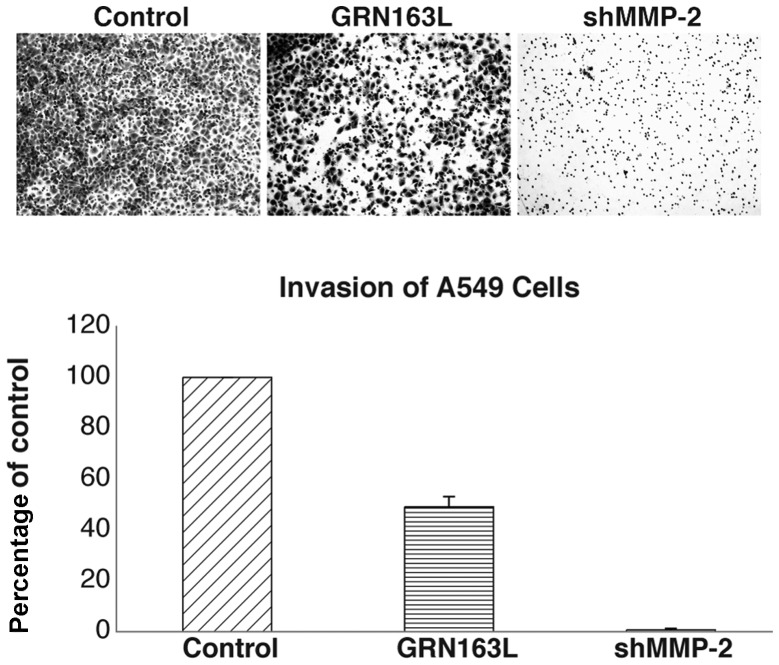
Invasion of A549 cells treated with GRN163L. A549 cells were treated with 1 *μ*M GRN163L for 24 h, then plated in Matrigel-coated invasion chambers for 22 h. Knockdown cells (shMMP-2) were used as the negative control (n=2).

When MMP-2 mRNA expression was determined by real-time PCR, a decrease in MMP-2 mRNA expression of ∼40% was observed in the A549 cells treated with GRN163L (Fig. 7). To determine whether this decrease was functional, we examined the motility/migration/invasive ability of the A549 cells treated with GRN163L. The cells were exposed to GRN163L for 24 h prior to plating on Matrigel-coated invasion chambers; the cells were then allowed to migrate/invade for 22 h. As shown in Fig. 8, GRN163L treatment decreased the invasive ability of the A549 cells by ∼50%, whereas the untreated cells were still able to invade. The results for the MM-treated control cells were similar to those of the untreated cells (data not shown). The MMP-2 knockdown efficiency was assessed using quantitative real-time PCR. The results were quantified following normalization to non-silencing shRNA. The percentage of MMP-2 shRNA knockdown cells was 28%. shMMP-2 knockdown cells were used as the negative control in the experiment (Fig. 8) demonstrating the significance of MMP-2 for the migration through the Matrigel-coated membrane.

## Discussion

Unregulated cell proliferation, escape from apoptosis, increase in tumor neovascularization (angiogenesis), migration, invasion and metastasis are all common features of various types of cancer (16). GRN163L is a telomerase template antagonist, that inhibits telomerase activity by binding to the template region of hTR. We discovered that GRN163L exerts additional effects, apart from the inhibition of telomerase, namely the disruption of cytoskeletal proteins, such as actin, tubulin, cytokeratin and α-actinin, as well as E-cadherin organization, and thus impairs cell adhesion and affects cell morphology. Using western blot analysis, we observed a significant decrease in actin, tubulin and E-cadherin expression in the GRN163L-treated cells, compared to the untreated control and MM oligonucleotide-treated cells (Fig. 2). Immunohistochemical staining also revealed that GRN163L disrupted the organization of 3 basic elements of the cytoskeleton: actin, tubulin and intermediate filaments. The altered cell morphology (i.e., rounding) observed in response to GRN163L treatment, may be a result of the disorganization of basic elements of the cytoskeleton, that are the key to sustaining the shape of the cell and structural scaffold. Of note, the MM control oligonucleotide (MM oligonucleotide sequence, 5′-Palm-TAGGTGTAAGCAA; GRN163L oligonucleotide sequence, 5′-Palm-TAGGGTTAGACAA) did not have any effect on cell morphology or cell adhesion (14). Since the MM control differs from GRN163L only by the lack of 3 contiguous guanine residues, it is possible that this motif is responsible for the altered cell morphology and adhesion phenotype upon treatment.

A similar reduction in migration and metastasis following treatment with GRN163L has previously been demonstrated in lung cancer cells using *in vivo* xenograft animal models (13,14), although the mechanisms underlying this effect were not elucidated in these studies. In this study, we demonstrate that the anti-adhesive effects of GRN163L, which may also contibute to the antimetastatic properties of this compound, are related to the disruption of cytoskeletal proteins, resulting in alterations in cell architecture and the intracellular relocalization of cytoskeletal elements. Consistent with this finding, Goldblatt *et al*(18) demonstrated similar results in MDA-MB-231 breast cancer cells; when GRN163L was added to the medium prior to cell attachment, it altered cell morphology, actin filament organization and focal adhesion formation.

Shin *et al*(19) demonstrated that actin disruption induced the phosphorylation of H2AX, a well-known double-strand break (DSB) marker, leading to G2 phase arrest and consequently resulting in the apoptosis of MCF-7 cancer cells. Based on these data, the authors suggested that actin disruption may be a potential candidate in the development of anticancer therapies for human cancers. Microtubules are also considered as important cellular targets for anticancer therapy, due to their key role in mitosis. Microtubule inhibitors such as taxanes, vinca alkaloids and epothilones, stabilize or destabilize microtubules, thereby suppressing microtubule dynamics required for proper mitotic function, effectively blocking cell cycle progression and resulting in apoptosis (20).

E-cadherin is critical for epithelial cell-cell adhesion. It is a well-known fact that, in order to be able to metastasize, cancer cells require attachment to a solid surface, in addition to their ability to proliferate and migrate (16). Our western blot analysis and immunostaining results support the hypothesis that the GRN163L-induced phenotypic changes may be attributed to alterations in the structural function of the treated cells. It is also possible that the downregulation of E-cadherin may reduce the attachment of cancer cells. In this case, the loss of adhesion may be due to the change in E-cadherin expression, resulting in the inability of cancer cells to attach. Additionally, the unattached ‘rounded’ cells lost their proliferative capacity and were reversibly arrested in the G1 phase of the cell cycle.

It has previously been reported that in NSCLC, the level of MMP-2 is increased in tumor cells, as well as in the peritumoral stromal tissues. Furthermore, MMP-2 expression has been reported to be an indicator of poor prognosis, associated with a worse overall survival (21). In this study, we demonstrated that GRN163L treatment led to a moderate decrease in MMP-2 expression in A549 lung cancer cells. Additionally, the migration/invasive ability of A549 cells through Matrigel decreased following a 24-h exposure to 1 *μ*M of GRN163L. These rapid effects of GRN163L were independent of telomerase activity and telomere length. In this study, to our knowledge, we demonstrate for the first time that GRN163L treatment decreases the migration/invasive capacity of tumor cells, possibly through the downregulation of MMP-2. These data suggest that GRN163L treatment following surgery and primary chemo/radiation therapy may prevent the invasion of residual cancer cells in NSCLC patients.

In conclusion, in the present study, we demonstrated that GRN163L altered the cell morphology due to the disruption of cytoskeletal elements and led to the loss of cell adhesion by decreasing E-cadherin expression. The cells treated with GRN163L were arrested in the G1 phase of the cell cycle. Furthermore, GRN163L inhibited the migration/invasion of A549 lung cancer cells through the downregulation of MMP-2. Based on these *in vitro* data, we hypothesized that residual circulating cancer cells present in the bloodstream, i.e., after tumor debulking surgery or chemotherapy, may be unable to attach and proliferate in the presence of GRN163L, due to the loss of adhesion properties and proliferative ability. Thus, the addition of a telomerase template antagonist to the anticancer therapy regimen may not only lead to a decrease in the growth of the primary tumor mass, but may also reduce the formation of distant metastases due to its nontelomerase-related effects.

## Figures and Tables

**Table I:** Primer sequences for real-time PCR analyses of G1 phase genes.

Target	Oligonucleotide sequences	Base pairs	GenBank accession no.
Cyclin D1	F: 5′-ATGAACTACCTGGACCGCTT-3′	142	NM_053056.2
	R: 5′-TCGGTGTAGATGCACAGCTT-3′		
Cdk4	F: 5′-GACCAGGACCTAAGGACATA-3′	146	NM_000075.2
	R: 5′-GTTCTCTGGCTTCAGATCTC-3′		
Cdk6	F: 5′-TTCACACCGAGTAGTGCATC-3′	122	NM_001259.5
	R: 5′-GAGGTTAGAGCCATCTGGAA-3′		
Cyclophilin A	F: 5′-AATGGCACTGGTGGCAAGTC-3′	219	NM_021130.3
	R: 5′-GCTCCATGGCCTCCACAATA-3′		

**Table II:** Primer sequences for real-time PCR analyses of matrix metalloproteinase-2 (MMP-2).

Target	Oligonucleotide sequences	GenBank accession no.
MMP-2	F: 5′-GTATCCATCGCCATGCTCC-3′	NM_004530
	R: 5′-AAGAACCAGATCACATACAGGATCA-3′	
GAPDH	F: 5′-GAGTCCACTGGCGTCTTC-3′	NM_002046.3
	R: 5′-GCATTGCTGATGATCTTGAGG-3′	
